# The dual activity of CaONPs as a cancer treatment substance and at the same time resistance to harmful microbes

**DOI:** 10.1038/s41598-023-49637-6

**Published:** 2023-12-22

**Authors:** Amr Awaad, Zakia A. Olama, Gehan M. El-Subruiti, Safaa M. Ali

**Affiliations:** 1 Department of Botany and Microbiology, Faculty of Science, Alexandria University, Alexandria, Egypt; 2 Chemistry Department, Faculty of Science, Alexandria University, Alexandria, Egypt; 3 Nucleic Acid Research Department, Genetic Engineering and Biotechnology Research Institute, City of Scientific Research and Technological Applications, New Borg El-Arab City, 21934 Alexandria Egypt

**Keywords:** Biochemistry, Biological techniques, Biotechnology, Cancer, Cell biology, Chemical biology, Microbiology, Diseases, Medical research, Materials science, Nanoscience and technology

## Abstract

Nanotechnology holds significant promise for the development of novel and necessary products that enhance human health. Pharmacology and nanotechnology have contributed to developing advanced and highly effective drugs for cancer treatment and combating microbial infections. The microbiological effectiveness against the variety of examined microorganisms was assessed using the time killer curve, scanning electron microscopy (SEM), MIC techniques, and the agar well diffusion method. SEM was utilized to enhance the analysis of the mechanisms underlying the bio-interface interaction and intracellular localization of calcium oxide nanoparticles (CaONPs). The MTT test was used to examine the cytotoxicity of CaONP anticancer activity in various cancer cells, including colon, breast, and hepatic cells. The efficacy of CaONPs as an anticancer medication was elucidated by analyzing the gene expression of both treated and untreated cancer cells. MIC and MBC of CaONPs against *Escherichia coli* and *Staphylococcus epidermidis* were 150, 150, 150, and 200 µg/ml, respectively. The MIC and MFC of CaONPs against *Candida albicans* were 200 µg/ml and 250 µg/ml, respectively. The IC50 values of various CaONPs vary depending on the type of cancer cells. The gene expression analysis of breast cancer cells undergoing treatment revealed the identification of several cancer-controlling genes, namely BAX, BCL2, P53, TERT, KRAS1, KRAS2, and RB1. The study demonstrated the notable antibacterial efficacy of CaONPs, highlighting their potential as cancer therapies.

## Introduction

Nanotechnology has had a significant positive impact on the biomedical and pharmaceutical industries. Nanoparticles (NPS) with the required physical and chemical properties can be produced. It is feasible to produce the appropriate quantity of metal oxide nanoparticles and further modify them with different chemical functional groups. Their functionalization enhances their compatibility for conjugation with ligands, cancer treatments, and biological substances (such as antibodies, nucleic acids, and peptides)^1,2^. Nanoparticles generate active oxygen, which mage the membrane cell wall through adhesion on the cell membrane, penetration through the membrane cell wall, and cellular internalization of nanoparticles. Metal oxide nanoparticles exhibit significant efficacy against disease-causing microorganisms. Several studies have also elucidated the cytotoxicity and genotoxicity of these nanoparticles. Calcium oxide (CaO), a metal oxide, has been widely used in bacterial and cancer research studies due to its nanoparticles’ high surface-to-volume ratio. This ratio allows for increased contact with bacteria and cancer cells, rendering it crucial in these investigations^3,4^. Reactive oxygen species (ROS), such as hydrogen peroxide (H_2_O_2_), hydroxyl radicals (OH)^−^, and peroxide (O_2_)^2−^, are produced during the mechanisms underlying these characteristics. These characteristics have led to the deterioration of mitochondria, the leakage of substances within cells, and the activation of genes related to oxidative stress^5–7^. *Escherichia coli*, the most prevalent Gram-negative opportunistic pathogen in humans, is responsible for causing stomach ulcers and symptoms such as nausea, vomiting, and diarrhea. The severity of these symptoms can range from mild and watery to severe and bloody^4,8,9^. One of the most common cutaneous resident bacteria, *Staphylococcus aureus* is also antibiotic-resistant^9–11^. *Candida albicans,* found in normal human mucocutaneous flora, are mainly responsible for septicemia and disseminated candidiasis, especially in patients with lymphoma, leukemia, and diabetes. *Candida albicans* also exhibit a significant prevalence of antifungal drug resistance^12^. Although only a few studies have reported the antibacterial properties of CaO, they show that CaO powder has significant promise as a bactericidal agent. It exhibits lower toxicity and has not been found to cause any endocrine disruption^13,14^. The biological applications of CaONPs, including their antibacterial and anticancer effects against cancer cells, as well as probable modes of action and gene expression, are highlighted in this study.

## Materials and methods

### Preparation of CaONPs

**Figure 1:**
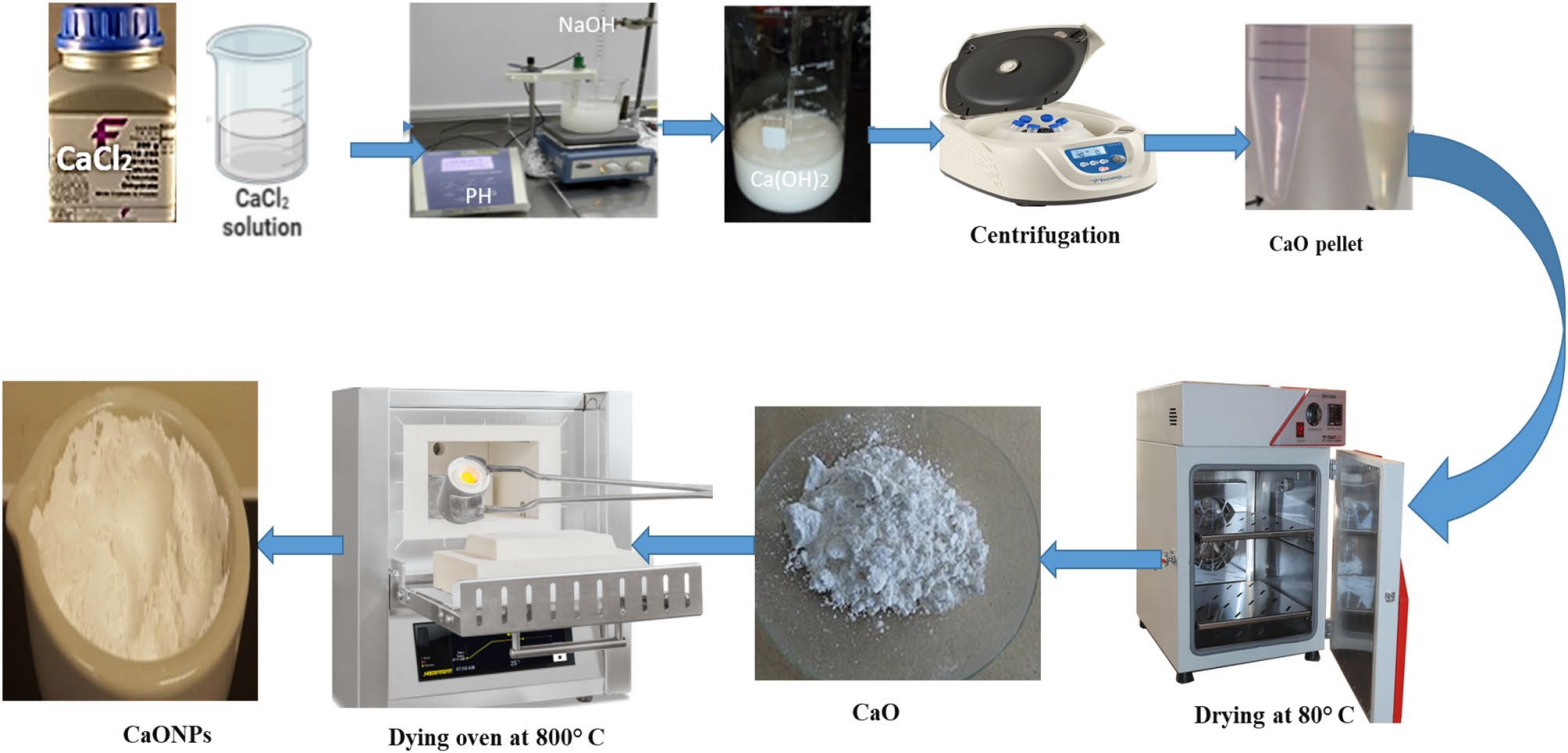
Direct thermal decomposition method for CaONP preparation.

CaCl_2._2H_2_O, NaOH, distilled water, and argon, with a purity of 99%, were used in the experiments. Precursor [Ca(OH)_2_] was prepared by addition of 1 and 2 M NaOH aqueous solution to a 0.5 M calcium chloride aqueous solution dropwise with vigorous stirring (1300 rpm) at 80 °C, while the inert gas (argon) was flowing on the solution surface. Over a period of time, the formation of a white solid of calcium hydroxide occurred, and the reaction stopped at pH = 11.2. The precipitate was then filtrated, rinsed five times with warm distilled water, and dried in the desiccator for several hours. A small portion of the produced powder was used for analysis, and the rest was calcined in a muffle furnace at 800 °C for 30 min under an N2 atmosphere at a heating rate of 5 °C/min (Fig. 1). The reactions that occurred in this work are shown in equations:

$$\begin{gathered} {\text{CaCl}}_{{2({\text{aq}})}} + 2{\text{NaOH}}_{{({\text{aq}})}} \to {\text{Ca(OH)}}_{{2({\text{s}})}} + 2{\text{NaCl}}_{{2({\text{aq}})}} \hfill \\ {\text{Ca(OH)}}_{{2({\text{s}})}} + 2{\text{NaCl}}_{{2({\text{aq}})}} + {\text{nH}}_{2} {\text{O}} \to {\text{nH}}_{2} {\text{O + }}2{\text{NaCl}}_{{2({\text{aq}})}} /{\text{Ca(OH)}}_{{2({\text{s,wet}})}} \hfill \\ {\text{Ca(OH)}}_{{2({\text{s,wet}})}} \to {\text{CaO}}_{{\text{(s)}}} + {\text{H}}_{2} {\text{O}}_{{\text{(g)}}}\uparrow \hfill \\ \end{gathered}$$

### CaONPs characterization

The infrared spectroscopy was conducted using the Infra-Red Bruker Tensor 37 instrument, which has a spectral range^15^ of 7500 to 370 cm^−1^. Additionally, the morphological characterization of CaONPs was determined using XRD and SEM. Zetasizer was used to measure the size of CaONPs^16^.

### Antimicrobial Activity Assay

#### Agar diffusion method of CaONPs

The antibacterial activity of the selected CaONPs was carried out using the well diffusion method. The CaONP samples stock solution (1000 µg/ml) and dilutions of the stock solution containing (50, 100, 150, 200, and 250 µg/ml) of CaONPs against bacteria (Gram-negative, gram-positive) and yeast. The agar diffusion technique was used to test the antimicrobial activity by preparing a standard inoculum of bacteria equivalent to 1.5 × 10^6^ CFU/ml of Gram-negative bacteria, 2 × 10^6^ CFU/ml of Gram-positive bacteria, and 2 × 10^5^ CFU/ml of yeast. A 0.5 McFarland standard inoculum was prepared, and 25 μl of it was applied to the surface of the Mueller Hinton Agar plate for bacteria and the Sabouraud dextrose agar plate for yeast^17^. The plate was kept at 4 °C for 1 h for compound diffusion. Plates inoculated with the test organism had 6-mm wells cut into the surface of the agar using a cork borer dipped in alcohol and flamed. After incubating the plates with bacteria for 24 h at a temperature of 37 ± 2 °C, 100 µl of antimicrobial solutions were added to the wells. The plates were then incubated with yeast for 72 h at a temperature of 22.5 ± 2 °C. The incubation period, the inhibition zone, and the diameters of any clear zones around the antimicrobial-containing wells were measured using calipers^18,19^.

#### Minimal inhibitory concentration (MIC) of CaONPs

An 80 μl mixture of sterile Müeller–Hinton broth, 20 μl of tween 80, and 100 μl of the CaONPs were diluted in a 96-well microtiter plate using a two-fold dilution method. Each well was inoculated with 100 μl of bacterial suspensions equivalent to 1.5 × 10^6^ CFU/ml, prepared using a 0.5 McFarland standard. The plates were covered and incubated at 35 ± 2 °C for 24 h. MIC was the lowest concentration of CaONPs that inhibited the growth of the bacterium. A 100 μl mixture of sterile Sabouraud dextrose broth and 100 μl of the CaONPs was diluted in a 96-well microtiter plate using a two-fold dilution method. Each well was inoculated with 100 μl of 0.5 yeast suspensions equivalent to 2 × 10^5^ CFU/ml. The plates were covered and incubated at 22.5 ± 2 °C for 48 h. MIC was the high concentration of CaONPs that inhibited yeast growth^20,21^.

#### Minimum bactericidal concentration (MBC) of CaONPs

After the estimation of the MICs, 20 μl aliquots from each well were plated onto Mueller Hinton Agar (MHA) plates and incubated at 35 ± 2 °C for 18 h. The MBC (minimal bactericidal concentration) was determined as the lowest dilution at which no bacterial growth was observed^22^.

#### Minimum fungicide concentration (MFC) of CaONPs

After estimation of the MICs, 20 μl aliquots from each well were plated onto Sabouraud dextrose agar plates and incubated at 22.5 ± 2 °C for 72 h. The lowest dilution not exhibiting yeast growth was recorded as the minimal fungicide concentration (MFC)^23–25^.

#### MIC index

The antimicrobial agent's MIC index (MBC/MIC) was calculated to determine whether it was bacteriostatic (MBC/MIC > 4) or bactericidal (MBC/MIC 4) against the growth of the tested bacteria. Bacteriostatic is defined as the range of MIC index values greater than 4 and less than 32. To determine if the antifungal impact was fungicidal or fungistatic, use the MFC/MIC or MIC index. The antifungal agent is considered fungicidal or fungistatic when the MFC/MIC ratio is below 2.0^26,27^.

#### Determination of bacterial time-kill curve

The time-kill curve was assessed to determine the optimum time for the CaONPs under test to kill the (Gram-positive, Gram-negative, and yeast). CaONps that demonstrated a bactericidal effect against the tested bacterium and mold were used, and the time-kill curve was plotted. A culture that was 16 h old was collected by centrifugation. The suspension was adjusted using the McFarland standard and was then further diluted in 0.85% saline to achieve approximately 1 × 10^6^ CFU/ml from bacteria suspension and 2 × 10^4^ CFU/ml for the yeast suspension. CaONps was added to aliquots of 1 ml Müeller–Hinton broth and Sabouraud dextrose broth based on their MIC values against the targeted bacteria and yeast. Subsequently, 1 ml of the inoculum was added. Further samples were taken from each tube to monitor by pour plate count at different time intervals (0, 2, 4, 6, 8, and 12 h)^28^.

#### Evaluation of CaONPs synergistic effect

The antibacterial activity of common antibiotics [Amoxicillin (Ax, 25 µg), Cefoperazone (CPZ, 75 µg), Penicillin (P, 5 µg), Cefoperazone/Sulbactam (SCF, 105 µg), Spiramycine (SP 100 µg)] combined with CaONPs was evaluated against *Staphylococcus aureus* and *E. coli*. The antibacterial activity of different CaONP contractions under test was evaluated against the selected pathogens. A standard inoculum of 1.5 × 10^6^ CFU/ml equivalent to 0.5 McFarland was prepared. Subsequently, 25 μl was applied to the surface of the Mueller Hinton Agar plate using a swab. The antibacterial activity was assessed using the disc diffusion method by adding disc paper in each concentration on a border, putting the saturated disc on the plate, and using different common antibiotics. The plates were kept for 1 h at 4 °C as a substitute for compound diffusion and then incubated for 24 h at 35 ± 2 °C. At the end of the incubation period, the inhibition zone diameters were recorded in millimeters^29^.

#### Scanning electron microscopic examination of the treated cells

The potential effect of CaONPs on the cell morphology of *E. coli* (gram-negative organism), *Staphylococcus aureus* (gram-positive organism), and *Candida albicans* (yeast) was determined with the aid of SEM. Bacterial cells and yeast cells were incubated with or without CaONPs for 6 h. The items were processed individually, undergoing three cycles of washing with a phosphate buffer solution at a pH of 7.4, followed by centrifugation at 4000 rpm. The pellets obtained were resuspended in the same buffer, and a thin smear was prepared on a glass slide, further fixed with glutaraldehyde (2.5%) for 2 h. Following fixation, a dehydration process was carried out using a gradient of ethanol concentrations ranging from 50 to 100%. The cells were dehydrated using liquid carbon dioxide. The desiccated cells were plated with gold using a sputter coater and subsequently examined by a scanning electron microscope (SEM) (JEOL JSM-6390LV) at the Electron Microscope Unit, Faculty of Science, Alexandria University, Egypt^30^.

### Anticancer

#### Cytotoxicity assay of calcium oxide nanoparticles

The MTT test, which relies on metabolically active cells converting the yellow tetrazolium salt-MTT into purple-formazan crystals, offers a quantitative assessment of live cells. 100 ml of RPMI 1640 and 2 × 10^5^ ml^−1^ of cells in each well are plated into 96-well plates and then developed for 24 h at 37 °C and 5% CO_2_. Subsequently, the media is extracted and replaced with a new medium incubated with various sample concentrations for 48 h. Each well was treated with 20 µl of MTT (3-4, 5-Dimethylthiazol-2-yl)-2, 5-Diphenyltetrazolium Bromide stock solution (5 mg/ml in PBS), which is then added and allowed to sit for 5 h. The medium is removed, and 200 μl DMSO is added to each well to dissolve the MTT metabolic product. The plate was shaken at 150 rpm for 5 min, and the optical density was measured at 560 nm. Untreated cells (basal) are used as a control of viability (100%), and the results are expressed as % viability (log) relative to the control. The optimal cell count for colon, hepatic, and breast cancer cell lines, obtained from the American Type Culture Collection (ATCC, USA), was determined through incubation for a specific period. Determination of the cell type should only be conducted once for each specific cell type^31^. Once the MTT molecule is exposed to live cells, it is enzymatically reduced to formazan, which is the basis of the MTT assay, a colorimetric viability test. The MTT molecule changes color as a result of the reduction. The absorbance indicates the amount of viable cells that remained after treatment with the CaONPs, and is compared to the absorbance of control cells that were not exposed to the CaONPs. Analysis of the results by proper software provides the IC50 (inhibition concentration; 50%) values and their statistical errors based on several repetitions of the measurement.

$$\% \;{\text{cell}}\;{\text{viability}} = \frac{Absorbance\;of\;treatment\;cells}{{Absorbance\;of\;control\;cells}} \times 100$$

#### Quantitative analysis of the oncogenes and proapoptotic gene expression

The MCF-7 breast cancer cells, obtained from the American Type Culture Collection (ATCC) in the United States, were chosen due to their previously established efficacy in inhibiting the proliferation of CaONPs. Total RNAs from MCF-7 cells treated and untreated with the investigated CaONPs were collected using the Gene JET RNA Purification Kit (Thermo Scientific, USA).

Real-time PCR was performed using the Verso 1-step RT-qPCR Kit, SYBR Green, and low ROX master mix. The CFX Connect Real-Time PCR System was utilized to subject the samples to a total of 40 amplification cycles, consisting of denaturation at 95 °C for 15 s, annealing at 60 °C for 30 s, and 72 °C for 30 s^32^. All amplifications were conducted in triplicate. The sequences of the primers (BAX, BCL2, P53, TERT, KRAS1, KRAS2, and RB1 gene) used in this study are presented in Table 1. Using the housekeeping gene B-actin, the ΔCT equation was utilized to calculate the difference in gene expression between treated and untreated cancer cells. The 2^−ΔΔCt^ method was used to calculate the relative fold change in the expression of these target genes in the treated cells.

**Table 1:** Primer list used in gene expression analysis.

Gene	Forward	Reverse
β-Actin	AAGCAGGAGTATGACGAGTCCG	GCCTTCATACATCTCAAGTTGG
BAX	CAAACTGGTGCTCAAGGCCC	GGGCGTCCCAAAGTAGGAGA
BCL2	CTGGTGGACAACATCGCCCT	TCTTCAGAGACAGCCAGGAGAAAT
P53	GCGTGTTTGTGCCTGTCCTG	TGGTTTCTTCTTTGGCTGGG
TERT	CGGAAGAGTGTCTGGAGCAA	GGATGAAGCGGAGTCTGGA
KRAS1	ACTGAATATAAACTTGTGGTAGTTGGACCT	TCAAAGAATGGTCCTGGACC
KRAS2	ACTGAATATAAACTTGTGGTAGTTGGACCT	CAAATCACATTTATTTCCTACCAGGACCAT
RB1	GACCCAGAAGCCATTGAAATCT	GGTGTGCTGGAAAAGGGTCC

### Statistical analyses

The data were presented as a mean standard deviation. *T*-test was used to compare the effects of CaONPs dispersion and free CaONPs on microbial or cancer cells. A value of 0.05 > p > 0.01 was considered significant for all tests. A p-value of < 0.01 was regarded as highly significant.

## Results and discussion

### Characterization of the calcium oxide nanoparticles

#### Microscopic observation

**Figure 2:**
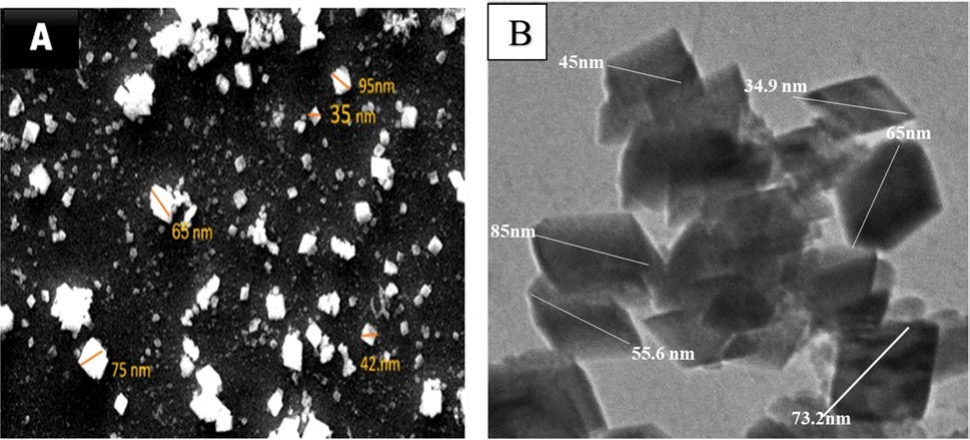
Scanning electron microscope (**A**), TEM images of CaONPs (**B**).

The CaONPs morphology was examined by scanning electron microscope (SEM) and Transmission Electron microscope (TEM). The average diameter of the CaONPs was obtained using Image-J software. The SEM images revealed that the homogeneous CaONPs had a fine diameter (35 and 95 nm) (Fig. 2A). The TEM images revealed that homogeneous CaONPs had a fine diameter (34.9 and 85 nm) (Fig. 2B). The diameter of prepared CaONPs was similar to the previous study^33^. All reflection peaks are easily correlated to the Fm-3 space group of a pure cubic phase of CaONPs (ICSD 75785). The X-ray wavelength is (1.54060 for Cu Ka1), and K is the so-called shape factor, which typically has a value of around 0.94. CaONPs crystallites are 55 nm in size on average.

### Antimicrobial activity

#### Agar well diffusion methods

**Figure 3:**
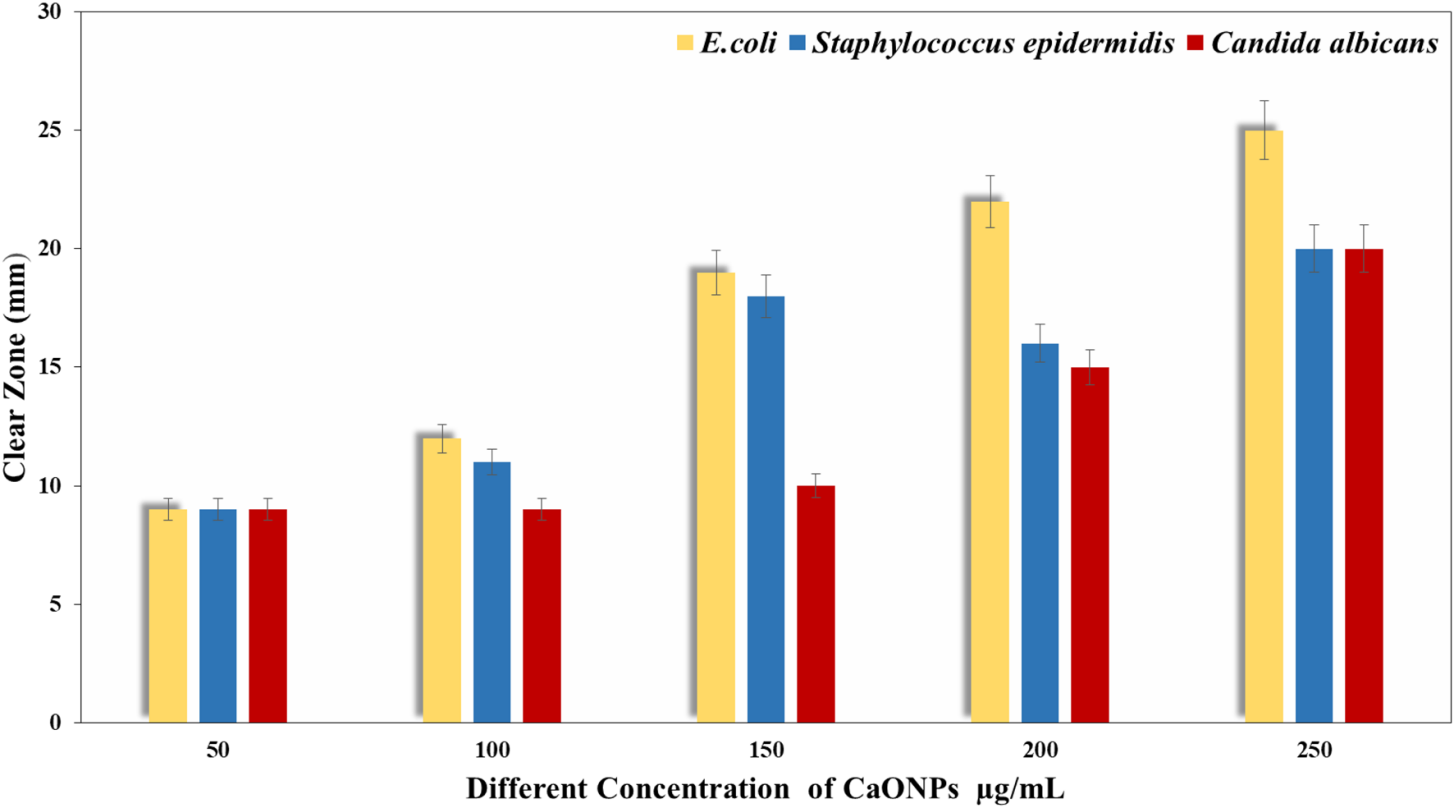
The antimicrobial activity of CaONPs (50–250 µg/ml) against different microbial cells using agar well diffusion methods.

The agar well diffusion technique was used to evaluate the antibacterial activity of CaONPs against different microbial strains^34^. The concentrations of CaONPs were (50, 100, 150, 200, and 250 µg/ml). Significant inhibition zone for *E. coli, Staphylococcus epidermidis,* and *Candida albicans* has been obtained in Fig. 3. The vast surface area of the CaONPs gives them exceptional antiseptic characteristics, while the smaller particle size makes the CaONPs more reactive with harmful microbes^35^. These nanoparticles may easily enter microbial cells because of their reduced size, which triggers an inhibitory mechanism inside the microbial cell^36^. A CaONPs that has entered a microbial cell induces cell death by distorting and destroying the cell membrane^34^. The inhibition zone for these microbes has been obtained at different concentrations of CaONPs (50–250 µg/ml). The inhibition zone increases by increasing the concentration of CaONPs.

#### Determination of MIC, MBC, MFC, and MIC index of CaONPs

The antibacterial effect of CaONPs against *Staphylococcus epidermidis*, *E coil*, and *Candida albicans* growth was verified by two established measures: minimum inhibitory concentration (MIC) and minimum bactericidal concentration (MBC)^37^. A comparative study of the MIC, MFC, and MBC values of different microbes was presented in Table 2.

**Table 2:** Antibacterial activity of CaONPs against different microbes.

Name of organism	Antimicrobial activity		
	MIC (μg/ml)	MBC and MFC(μg/ml)	MIC/MBC INDEX
*Staphylococcus epidermidis*	150	200	0.75
*E. coli*	200	200	1
*Candida albicans*	250	250	1.0

**Figure 4:**
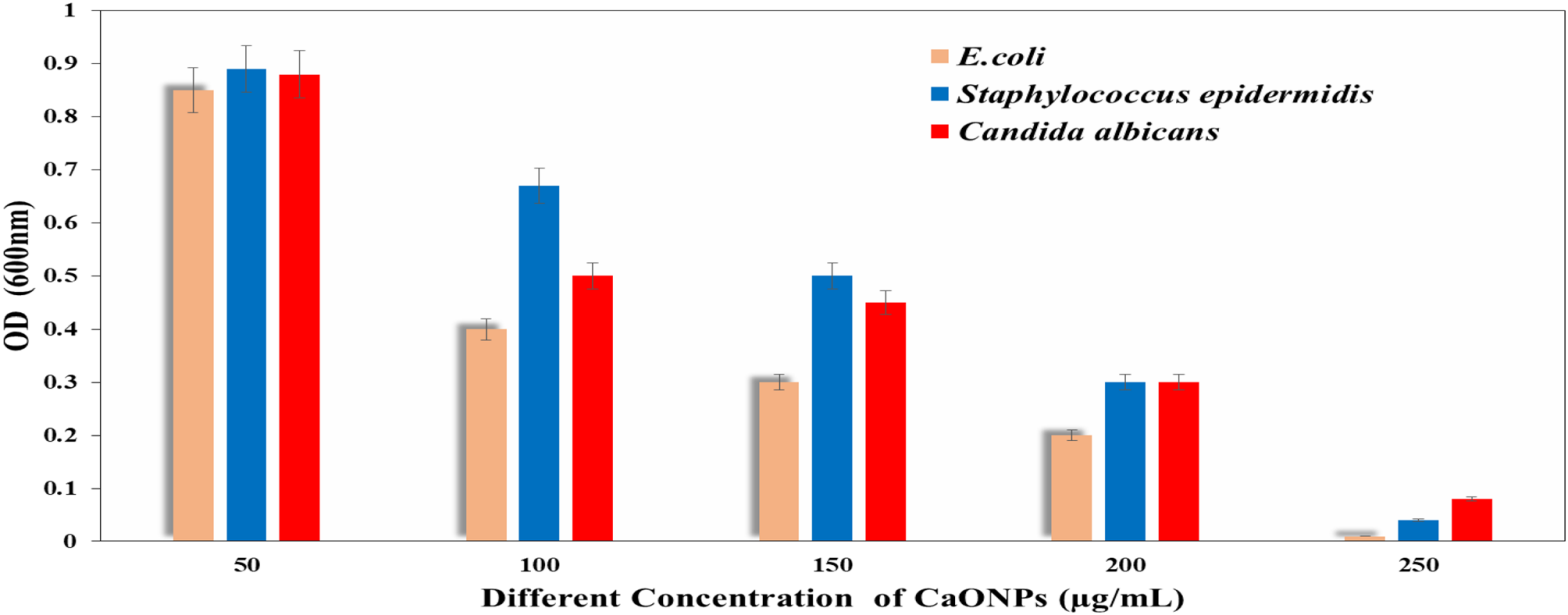
The antimicrobial activity of CaONPs against the bacterial strains i.e. *Escherichia coli*, *Staphylococcus epidermidis,* and *Candida albicans* by using different concentrations of CaONPs.

**Figure 5:**
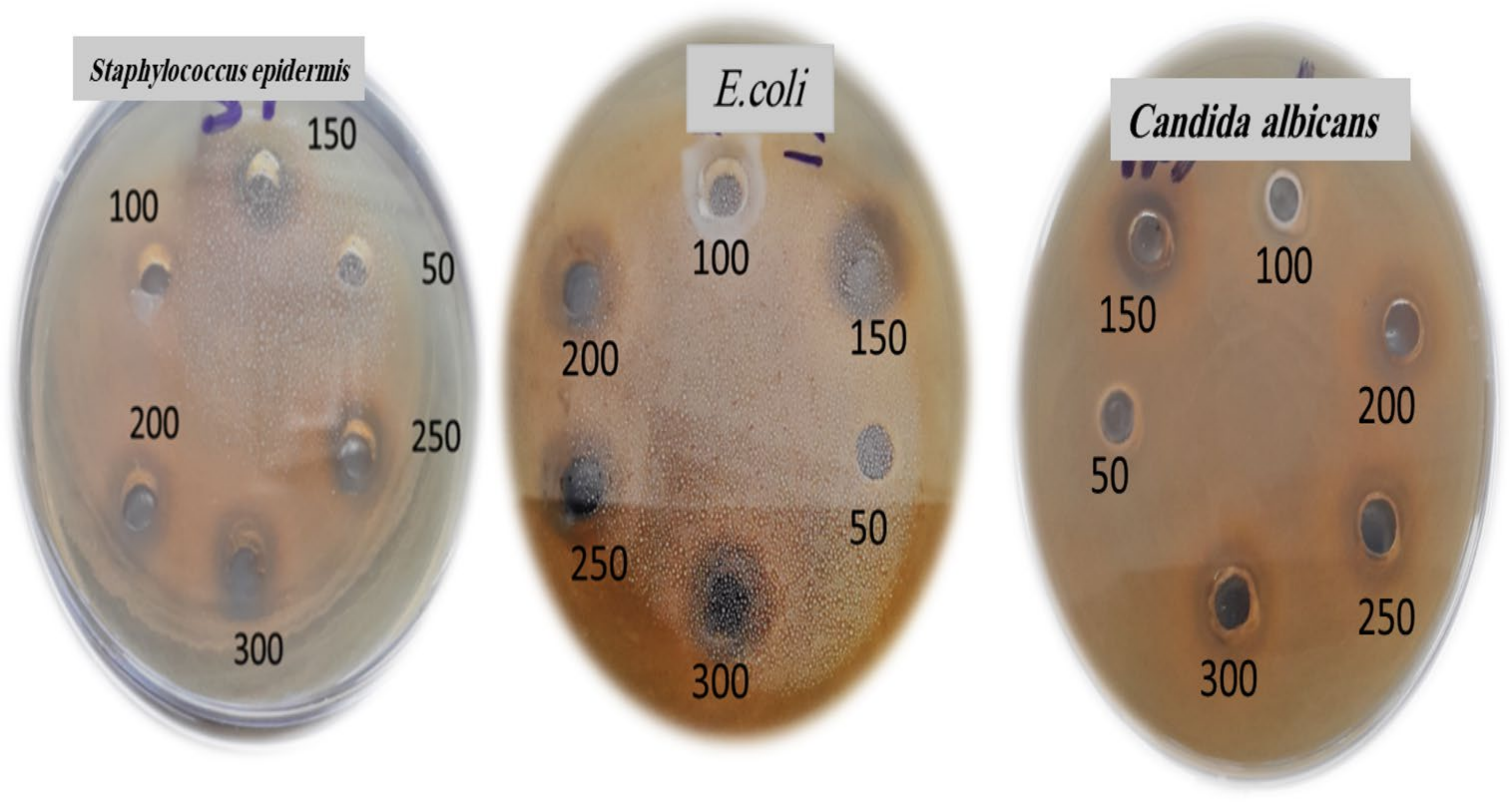
Antibiotic sensitivity test of CaONPs against the tested bacterial strains.

The antibacterial activity of CaONPs was studied against *E. coli, Staphylococcus epidermidis,* and *Candida albicans* using optical density measurements (Figs. 4 and 5). The results revealed that in the absence of CaONPs (control experiments), the optical density of cell suspension was found to be increased with time, and by increasing the concentration of CaONPs, the optical density of the microbe decreased^38^.

#### Time-kill assay

**Figure 6:**
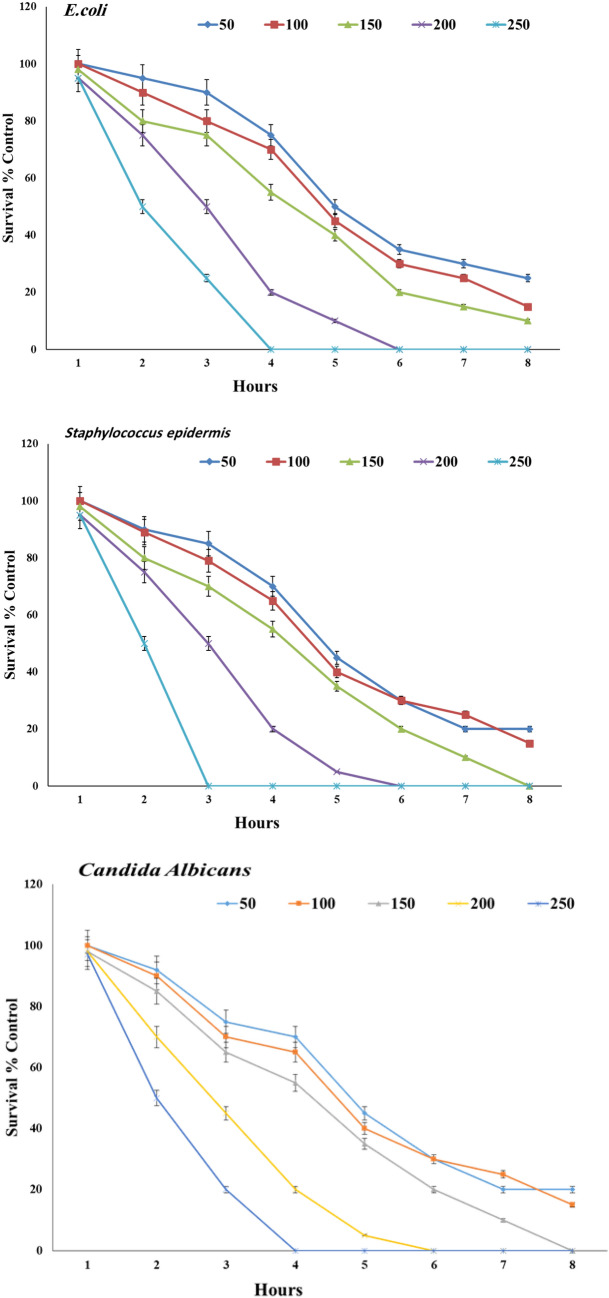
Reduction of the microbial population (*Escherichia coli*, *Staphylococcus epidermidis,* and *Candida albicans*) with time when treated with different concentrations of CaONPs (50, 100, 150,200, and 250 µg/ml).

The time-kill activity of (*Escherichia coli*, *Staphylococcus epidermidis,* and *Candida albicans*) pathogens is shown in (Fig. 6). The reduction in the number of CFU/ml was ≥ 3 Log units (99%). The bactericidal endpoint of CaONPs for *E. coli* was reached after 4 h incubation using (250 μg/ml), and 6 h incubation at (200 μg/ml). *Staphylococcus epidermis* was killed after 6 h of incubation at (200 μg/ml), and (250 μg/ml). *Candida albicans* were killed after 4 h at (200 μg/ml), and (250 μg/ml). CaONPs could be used as universal antimicrobial substances due to their strong biocidal effect against microorganisms, which have been used over the past decades to prevent and treat various diseases^39^. Recently, non-hazardous CaONPs can easily be synthesized using a cost-effective method and tested as a new type of antimicrobial agent^37^.

#### Evaluation of the synergistic effect of common antibiotics and CaONPs

**Figure 7:**
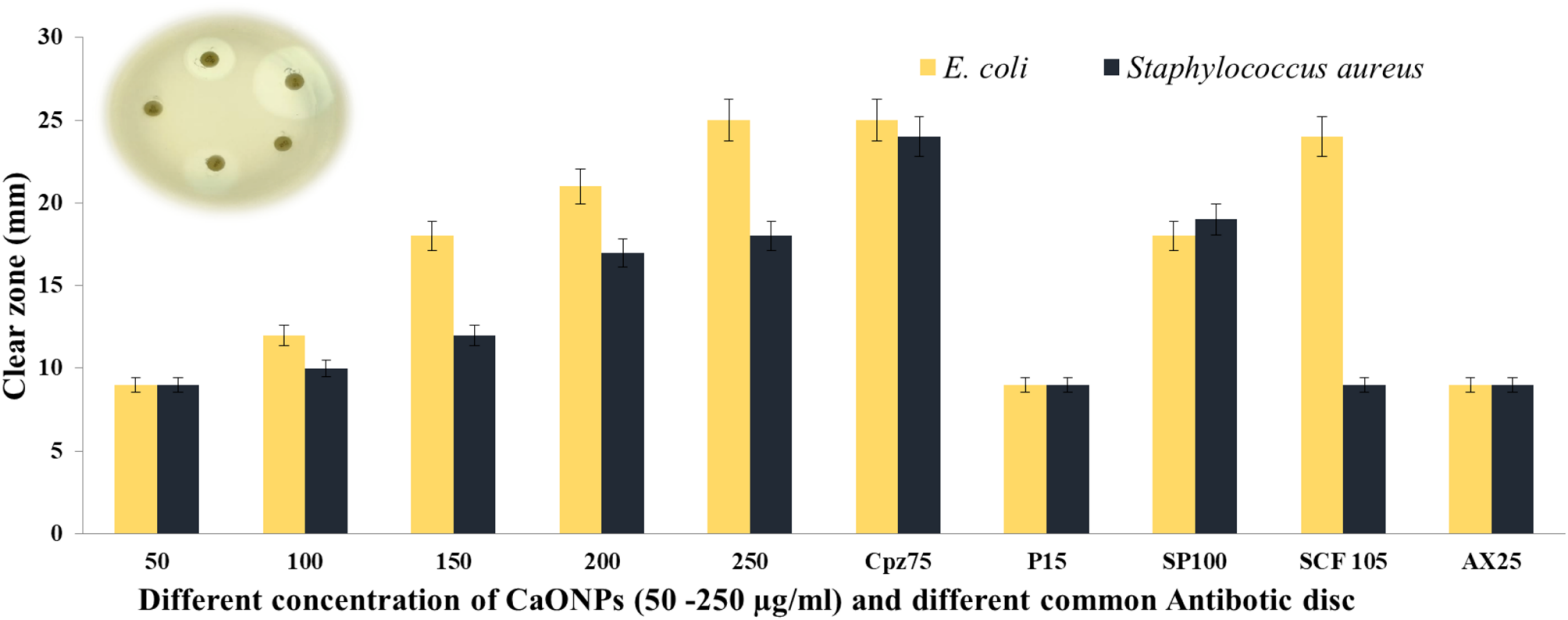
Evaluation of the synergistic effect of common antibiotics and CaONPs against *Staphylococcus aureus* and *E. coli.*

The study examined the combined impact of common CaONPs on *Staphylococcus aureus* and *E. coli*^40^, as depicted in (Fig. 7)*.* The CaONPs have antibacterial activity similar to some antibiotics. In addition, the inhibitory effects of various CaONP concentrations on the growth of *Staphylococcus aureus* and *Escherichia coli* were evaluated. CaONP antimicrobial activity was then compared to commonly used antibacterial agents, including Amoxicillin, Cefoperazone, Penicillin, Cefoperazone/Sulbactam, and Spiramycine. The optimal concentration at which CaONPs exhibit the most pronounced concentration effect on *Staphylococcus aureus* and *E. coli* is 250 µg/ml, which is comparable to the concentration of Spiramycine (SP 100)^41^.

#### Microscopic observations

**Figure 8:**
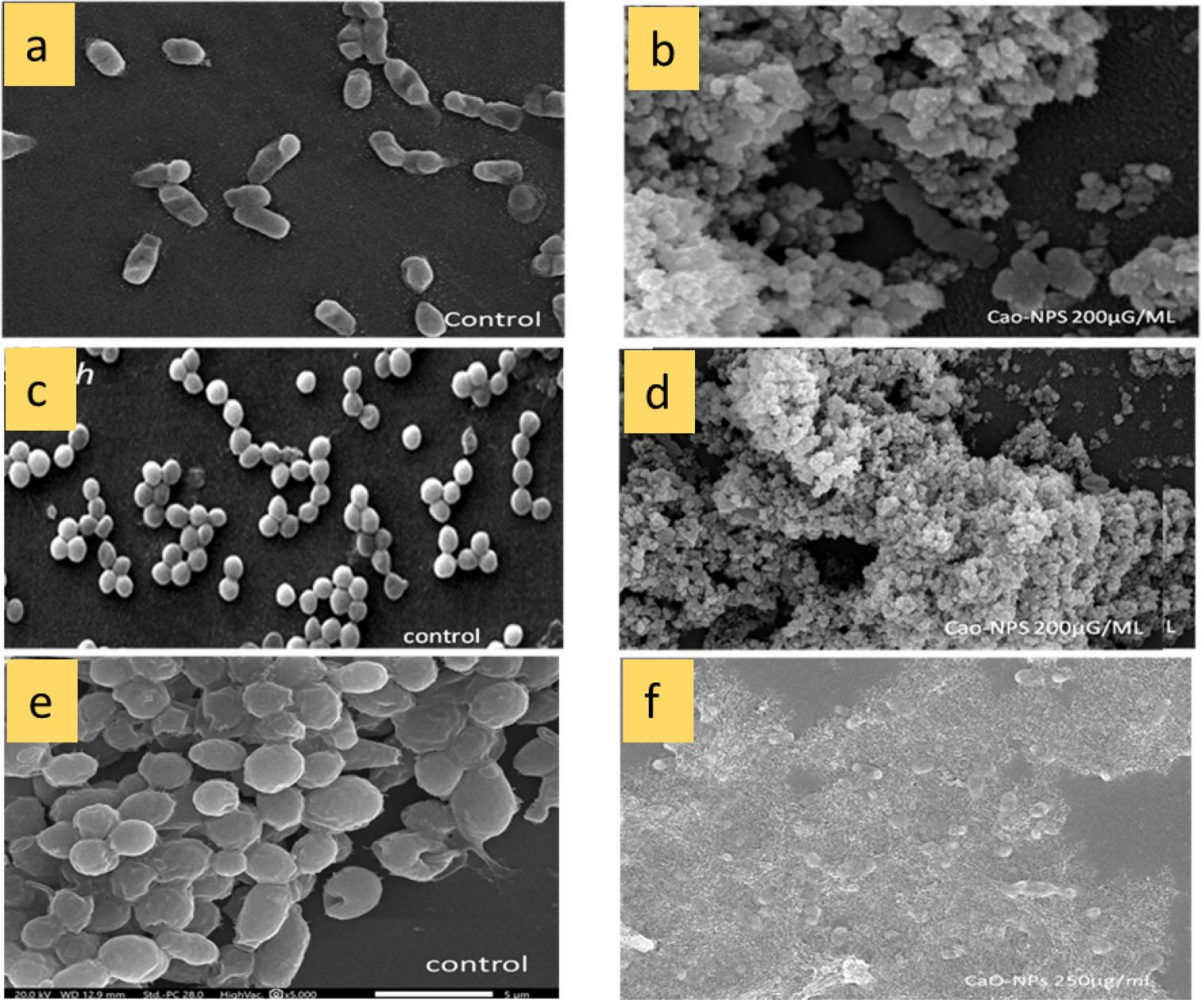
Scanning electron microscopic image of *E. coli* control and treatment by addition of 200 µg/ml CaONPs (**a**,**b**), *Staphylococcus aureus* control and treatment by addition of 200 µg/ml CaONPs (**c**,**d**), and *Candida albicans* control and treatment by addition of 250 µg/ml CaONPs (**e**,**f**).

The size and shape of *E. coli* cells exhibited irregularities, along with the occurrence of clamping, as a result of the concentration of CaONPs. Following the addition of 200 µg/ml of CaONPs, a limited number of cells were detected, exhibiting irregular morphology, and most of the cells were found lysed. Figure 8a,b shows a scanning microscopic image of *Staphylococcus aureus* after adding CaONPs. Following the introduction of a 200 µg/ml solution of CaONPs, a significant proportion of the cells exhibited elongation and irregular morphology, as depicted in (Fig. 8c,d). After 250 µg/ml of CaONPs were added, very few well-structured cells were found in *Candida albicans* (Fig. 8e,f). The optical inspection of bacterial specimens and *Candida albicans* was performed by phase contrast microscopy to detect the variation of the transparent biological object into the amplitude variation. All the microbial strains were found to lose their cell structure integrity after treatment with CaONPs. A spherical shape was observed due to the inhibition of cell division^42–44^.

### Evaluation of CaONPs as anticancer

#### Cytotoxicity

**Figure 9:**
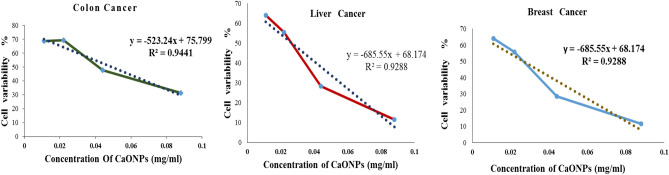
Cytotoxic effects of CaONPs on human colon cancer, human hepatic cancer, and breast cancer cell line. Each value represented is the mean ± SD of three independent experiments.

**Figure 10:**
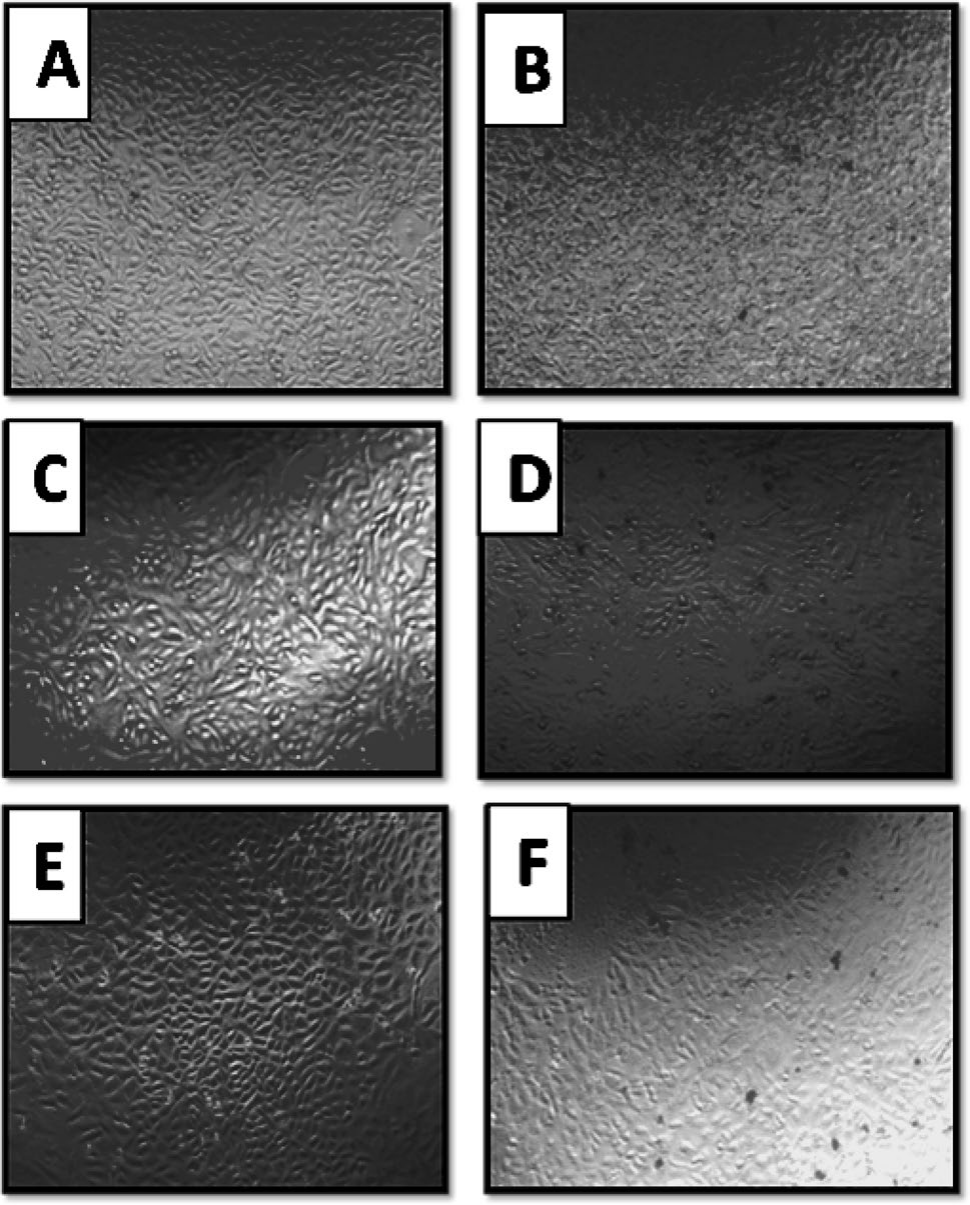
Bright field microscopy of colon cancer cells (**A**,**B**) hepatic cancer cells (**C**,**D**) and breast cancer cells (**E**,**F**) on treatment with nanoparticles at IC_50_. Control (**A**,**C**,**E**), CaO nanoparticle (**B**,**D**,**F**).

The CaONPs were screened for anticancer activity in the HT29 cell line. The nanoparticles were tested for activity in concentrations ranging from 11, 22, 44, 88, 176, and 350 µg/ml of the tested nanoparticles (Fig. 9). CaO nanoparticles showed concentration-dependent anticancer activity in HT29. Calcium nanoparticles have antitumor activity against breast cancer cells (MCF 7), Hep-G2 (liver cancer cells), and Colon Cancer. Recent studies have shown that Calcium nanoparticles can inhibit angiogenesis, a process that plays a crucial role in the growth and spread of cancer cells. The anticancer activity was due to the intrinsic property of calcium nanoparticles, which interact selectively with heparin-binding glycoproteins and inhibit their activity. It has been reported that CaONPs have antitumor activity against colon cancer. It had an IC_50_ value of 50 µg/ml^45,46^. It has been reported that Calcium oxide nanoparticles have antitumor activity against breast cancer cells (MCF 7) with an IC_50_ value of 25 µg/ml and antitumor activity against cancer cells Hep-G2 (liver cancer cells) with an IC_50_ value of 74 µg/ml (Fig. 10). The morphological changes of the cell line, on the addition of IC_50_ concentration of CaONPs, were recorded using an inverted phase-contrast tissue culture microscope (Olympus CKX41 with Optika Pro5 CCD camera). The changes in the morphology of the cells, such as rounding or shrinking of cells at varying degrees, are visible in the images and indicate cytotoxicity. The concentration of samples required for 50% of inhibition (IC_50_) was calculated. The high cytotoxic effect of nanoparticles is due to their high attraction towards biological macromolecules and effortless permeability to the cellular barriers. Some studies reported that nanoparticles cause cytotoxicity via reactive oxygen species. It causes damage to the cellular component by intracellular oxidative stress and finally leads to death. Through intracellular oxidative stress, it damages the cellular component and ultimately results in death. In a study, a carcinoma cell line treated with CaONPs shows vast anticancer activity and is supposed to create a major mark in cancer therapy^47,48^.

#### Quantitative analysis of the oncogenes and proapoptotic genes expression in the breast cancer cells (MC7)

**Figure 11:**
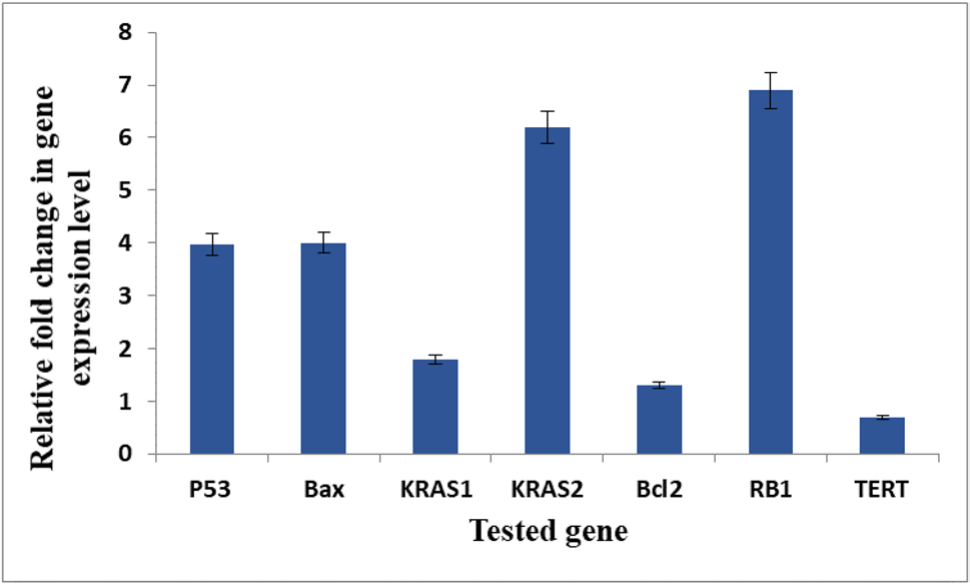
Oncogene and apoptotic gene expression in breast cancer cells treated with CaONPs (values are provided as mean SEM and different symbols indicate significance at (p < 0.05).

The qRT-PCR analysis revealed that the expression levels of essential genes associated with both cell proliferation and cell death were nearly equivalent in the MC7 cells that were treated with CaONPs (Fig. 11). Recent findings have revealed a reduction in BCl2 and TERT expression by approximately 1.5 and 0.7 folds, respectively. In the MC7 cells that underwent treatment, there was a nearly equal expression of key genes associated with both cellular growth and apoptosis^49^. The first member of the Bcl-2 family of regulator proteins, which control cell death (apoptosis) by either inducing (pro-apoptotic) or inhibiting (anti-apoptotic), is Bcl-2 (B-cell lymphoma 2), which is encoded in humans by the BCL2 gene. It was the first regulator of apoptosis found in an organism. The pro-apoptotic proteins in the BCL-2 family, including Bax and Bak, normally act on the mitochondrial membrane to promote permeabilization and release of cytochrome c and ROS, which are important signals in the apoptosis cascade^50,51^.

CaONPs are capable of decreasing P53, Bcl2, TERT, and Bax genes. The p53 delivery system could apply to a wide range of cancers, including those of the breast, colon, lung, and head and neck. p53 is involved in many mechanisms relating to cancer progression and treatment^52^. Activation of p53 typically occurs in response to DNA damage, cellular stress (including oncogene activation), and chemotherapeutic agents and often results in cell cycle arrest and/or apoptosis^53^. Apoptosis controller The human BAX gene encodes the protein known as BAX, or BCL-2-like protein 4. BCL2 family members regulate a wide range of cellular processes by forming hetero- or homodimers and acting as pro- or anti-apoptotic agents^54^. The expression of BAX is upregulated by the tumor suppressor protein p53, and BAX is involved in p53-mediated apoptosis. As a component of the cell's stress response, the transcription factor p53 activates and controls many downstream genes that are targeted, including BAX^55^. On the other hand, Telomerase activity via TERT expression has an impact on telomere length and can be a useful marker in the diagnosis and prognosis of various cancers and a new therapy approach^56^. Also, CaONPs are capable of increasing KRAS1, KRAS2, and RB1^50^. Since GTP has a greater intracellular concentration than GDP, KRAS is activated when a guanosine exchange factor (GEF) protein removes GDP from the nucleotide-binding site. This ultimately leads to GTP binding. Inactivation of the active KRAS occurs upon GTP hydrolysis to GDP. Mutated (changed) forms of the KRAS gene have been found in some types of cancer, including non-small cell lung cancer, colorectal cancer, and pancreatic cancer^57^. These changes may cause cancer cells to grow and spread in the body RB1 (The retinoblastoma gene) is a tumor suppressor gene that was first discovered in a rare ocular pediatric tumor called retinoblastoma (RB)^58^. The RB1 gene is essential for normal progression through the cell cycle and exerts part of its function through the family of transcription factors (E2F) and many other intermediaries^59^. In the absence of normal RB1, genomic instability and chromosomal aberrations accumulate, leading to tumor initiation, progression, and ultimately metastasis^60^.

The present study showed that up-regulation of these genes by CaONPs could increase the production of ROS and oxidative stress. It also supported that the Nano toxicity mechanism could correlate with active oxygen production, oxidative stress, apoptosis, and antioxidant defense mechanisms^61^. Though CaONPs can be used in anticancer therapy and may be used in combination with the anticancer drug, this formulation proves to be better than the existing ones, as the drug is loaded onto the nanoparticles, which makes the therapy less troublesome. It can be concluded that drug loading on CaONPs is a novel and effective approach towards cancer management, with better targeting of the highly toxic chemotherapeutic drugs, thus producing fewer side effects.

## Conclusion

Several studies have been conducted to examine the potent biological antibacterial properties of CaONPs, as well as their cytotoxic effects on cancer cell lines such as HepG2, Colon, and Breast. The findings of the present study indicate that CaONPs exhibit promising characteristics as therapeutic agents in the treatment of cancer and microbial infections. Additionally, it is suggested that CaONPs possess beneficial properties for utilization in various biomedical applications. Among the results obtained was that it has an excellent therapeutic effect on various cancers, specifically on breast cancer, for which the extent of the effect of the therapeutic substance on the cells and genes controlling the process was studied. This study indicates that when removing a tumor, the surgeon needs to use antibiotics to help the wounds heal.

It is recommended that further research be conducted utilizing animal experimental models to investigate the cytotoxicity and antimicrobial activity of the synthesized CaONPs.

## Data Availability

This published article contains all of the data that were examined throughout this investigation.
